# Single-Cell Analysis of RNA Virus Infection Identifies Multiple Genetically Diverse Viral Genomes within Single Infectious Units

**DOI:** 10.1016/j.chom.2015.09.009

**Published:** 2015-10-14

**Authors:** Marine Combe, Raquel Garijo, Ron Geller, José M. Cuevas, Rafael Sanjuán

**Affiliations:** 1 Instituto Cavanilles de Biodiversidad y Biología Evolutiva, Universitat de València, C/ Catedrático José Beltrán 2, 46980 Paterna, Valencia, Spain

## Abstract

Genetic diversity enables a virus to colonize novel hosts, evade immunity, and evolve drug resistance. However, viral diversity is typically assessed at the population level. Given the existence of cell-to-cell variation, it is critical to understand viral genetic structure at the single-cell level. By combining single-cell isolation with ultra-deep sequencing, we characterized the genetic structure and diversity of a RNA virus shortly after single-cell bottlenecks. Full-length sequences from 881 viral plaques derived from 90 individual cells reveal that sequence variants pre-existing in different viral genomes can be co-transmitted within the same infectious unit to individual cells. Further, the rate of spontaneous virus mutation varies across individual cells, and early production of diversity depends on the viral yield of the very first infected cell. These results unravel genetic and structural features of a virus at the single-cell level, with implications for viral diversity and evolution.

## Highlights

• Genetic diversity is found within viral units infecting an individual cell
• Viral particles within the same infectious unit interact genetically and functionally
• The production of viral genetic diversity is marked by widespread cell-to-cell variation
• Single-cell virus yields determine the early production viral genetic diversity

## Graphical Abstract

**Figure:**
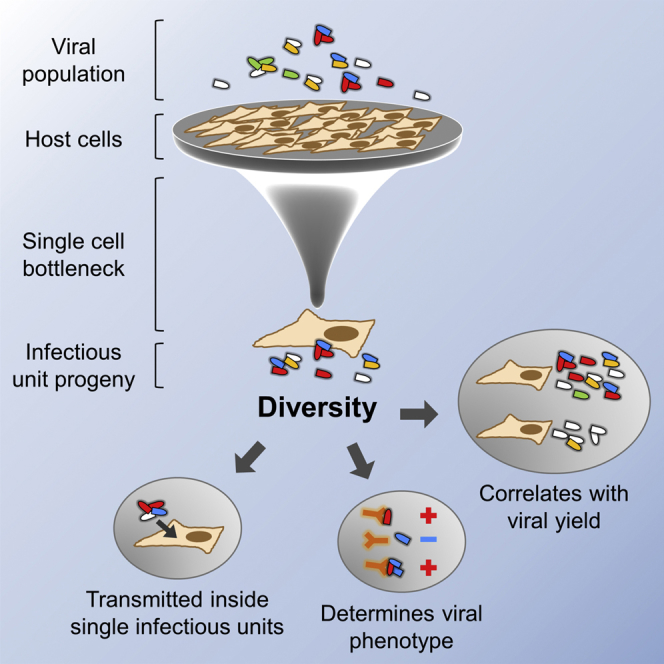

## Teaser

It is assumed that single virions constitute viral infectious units. Combe et al. combine single-cell isolation with ultra-deep sequencing to show that individual virus infectious units are constituted by multiple genetically diverse viral genomes. Thus, natural selection acts on pools of variants linked by their co-localization in the same cell.

## Introduction

Molecular and cell biology processes have been traditionally studied by averaging the behavior of large numbers of cells. However, it is becoming increasingly recognized that the study of variability among individual cells is important for understanding the regulation of gene expression, cellular differentiation, organismal development, or the evolvability of unicellular organisms (Eldar and Elowitz, 2010; Pelkmans, 2012; Raj and van Oudenaarden, 2008). Several observations suggest that cell-to-cell variation also has important implications for the outcome of viral infections. For instance, even under constant stimuli, the activation of interferon pathways varies significantly among individual cells (Rand et al., 2014). Stochastic cell-to-cell variability in viral infections has been demonstrated for several animal viruses, and its analysis has contributed to clarifying central aspects of the viral lifecycle, such as the intracellular determinants of viral fitness, or viral replication mechanisms (Schulte and Andino, 2014; Schulte et al., 2015; Timm and Yin, 2012; Zhu et al., 2009).

Genetic diversity is a key determinant of the ability of many viruses to escape natural immunity and vaccination, evolve drug resistances, and cause emergent diseases (Andino and Domingo, 2015; Duffy et al., 2008; Elde et al., 2012; Lauring et al., 2013; Pepin et al., 2010). However, the production of viral genetic diversity at the single-cell level remains largely uncharacterized. Although millions of cells are infected daily within each individual host, typically only a few viral particles succeed in being transmitted, such that new infections can be started from as little as one target cell (Abel et al., 2015; Gutiérrez et al., 2012). These drastic population bottlenecks are believed to represent a major sieve for viral diversity, to reduce the efficacy of natural selection, and to favor the accumulation of deleterious mutations in viral genomes by random genetic drift (Chao, 1990; Escarmís et al., 2006). The ability of viruses to maintain or replenish genetic diversity despite repeated transmission bottlenecks should thus be critical for the successful establishment of new infections and for long-term virus survival.

Here, by combining single-cell isolation with ultra-deep sequencing, we have determined the effects of single-cell bottlenecks on viral genetic diversity. As a model system, we have used vesicular stomatitis virus (VSV), a prototypical non-segmented negative-strand RNA virus belonging to the family Rhabdoviridae. Analysis of genome sequences from 881 plaques derived from 90 individual infected cells showed that VSV rapidly creates genetic diversity after the bottleneck. Furthermore, several genome variants can be delivered simultaneously to individual cells, thus favoring the maintenance of pre-existing sequence polymorphisms even under extreme bottlenecks. Particle aggregation defines a type of linkage between alleles, determined by their co-localization within the same infectious units and cells. As a consequence, natural selection can sometimes act on pools of co-transmitted variants, resulting in the concealment of deleterious or lethal mutations by *trans*-complementation with co-infecting genomes, or in the exposure of high-fitness alleles to purifying selection. Furthermore, we show that the rate at which new spontaneous mutations are produced varies among cells, and that the yield in the very first infection cycle correlates with the short-term ability of the virus to create diversity. Finally, we also found that some individual cells are infected with variants with increased rates of spontaneous mutation, thus contributing disproportionately to the production of genetic diversity relative to other cells.

## Results

### Effect of Single-Cell Bottlenecks on Viral Diversity

**Figure 1:**
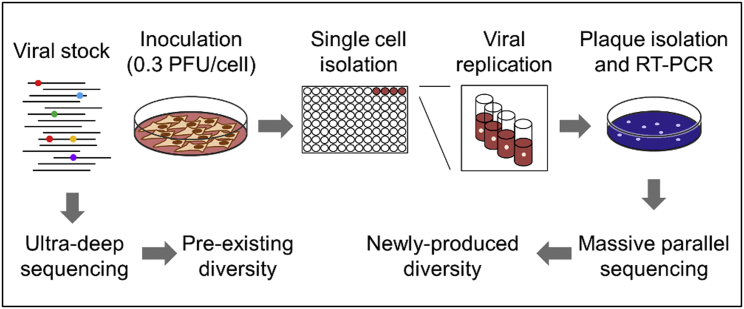
Experimental Setup for Sequencing Single-Cell Bottlenecked Viruses BHK cells were inoculated with approximately 3 × 10^4^ PFUs of a VSV stock at an MOI of 0.3 PFU/cell, and individual cells were transferred to separate culture wells with a micromanipulator. After overnight completion of the viral infection cycle, supernatants were plated in solidified medium and single, isolated plaques (viral progeny) were picked and used for SOLiD massive parallel sequencing. The viral stock was subject to Illumina ultra-deep sequencing to detect polymorphisms present in the inoculum (parental sequence variants). Polymorphisms present in plaque sequences but not detected in the inoculum constitute likely spontaneous mutations arising after the single-cell bottleneck.

VSV was recovered from an infectious cDNA clone, passaged four times in baby hamster kidney (BHK) cells, and frozen to obtain a viral stock. Each of these initial transfers was seeded with approximately 10^4^ plaque-forming units (PFUs) at a multiplicity of infection (MOI) of 0.1 PFU/cell, thus allowing for the completion of approximately two infection cycles per passage, during which genetic diversity was allowed to accumulate. Using this viral stock, we combined single-cell isolation and high-throughput sequencing to assess the effects of single-cell bottlenecks on viral genetic diversity (Figure 1). The viral stock diversity was determined by Illumina paired-end ultra-deep sequencing with an average coverage of 74,150 reads per base and > 10,000-fold coverage in 97.5% of the viral genome (Figure S1). In total, 197 single-nucleotide polymorphisms (SNPs) were detected at population frequency > 0.1% (Table S1), the empirically determined detection limit of this sequencing technology (Figure S2). To ascertain the effects of single-cell bottlenecks on viral diversity, approximately 3 × 10^4^ PFUs of the stock were used for inoculating BHK cells at MOI of 0.3 PFU/cell, and micromanipulation was used to isolate 350 individual infected cells within 2 hr post inoculation, a time preceding the release of viral progeny (Timm and Yin, 2012). After 24 hr incubation in separate wells, supernatants were titrated by the plaque assay to determine the viral yield per cell. We observed a productive infection (> 100 total PFUs) in 95 of the 350 cells (27%), consistent with the MOI used. Therefore, according to the principles underlying the Poisson distribution, 85% of the cells should be infected by a single PFU, and 15% by two or more PFUs. Since it was not technically feasible to reliably analyze the genetic diversity produced by the virus within the single infected cells, we sought to quantify diversity in plaques formed by the viruses released from the single cells. Given that plaque development requires approximately 24 hr and that VSV completes an infection cycle every 12 hr under these conditions (Cuevas et al., 2005), this approach allowed us to focus in the two viral generations following the initial single-cell infection. Virus-containing supernatants from individual cells were used for plaque assays, and 7–10 plaques from each cell were picked and subjected to SOLiD massive parallel sequencing. In total, we obtained the full-length sequence of 881 plaques derived from 90 individual cells. Each plaque was sequenced with an average coverage of 179.4 reads per base and a minimum coverage of 10 reads in 99.7% of the genome (Figure S3). Only variants present at > 20% frequency within plaques were considered, thus discarding sequencing errors or mutations produced during the late stages of plaque growth. Comparison with the consensus sequence of the virus used for inoculation revealed 532 SNPs in the 881 plaques sequenced. Then, to ascertain whether these SNPs were spontaneous mutations produced in the individual infected cells and its immediate progeny or, in contrast, were parental sequence variants (i.e., already present in the inoculum), we compared these SNPs with those found in the viral stock by ultra-deep sequencing. Of the 532 SNPs, 36 were found in the viral stock and were classified as parental variants (Table S2).

### Pre-existing Viral Genetic Diversity Delivered to Individual Cells

**Figure 2:**
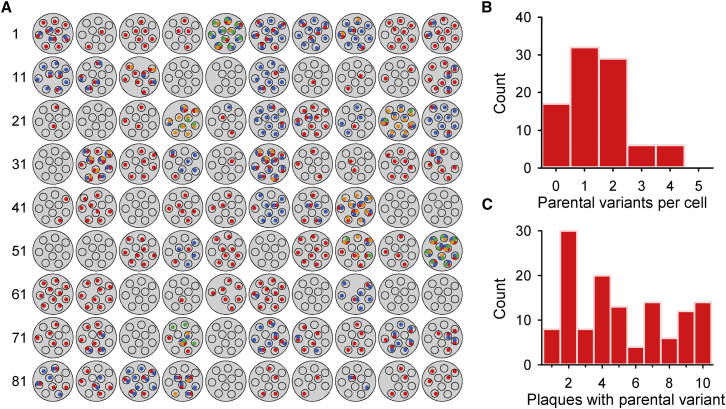
Parental Genetic Variants Delivered to Individual Cells (A) Parental sequence variants found after each single-cell bottleneck. Large gray circles depict individual cells labeled with numbers, and smaller circles correspond to the 7–10 plaques sequenced for each cell. The 36 parental variants are shown with colored dots inside each plaque. Colors are used for distinguishing different variants found among plaques derived from the same initial cell. Use of the same color in different cells is not meant to indicate a common sequence variant. (B) Distribution of the number of parental sequence variants found in the 7–10 plaques derived from single cells. (C) Distribution of the number of plaques derived from the same cell that contained a given parental variant. The list of parental sequence variants found in the inoculum is provided in Table S1 and the list of parental variants found in plaques derived from single cells is provided in Table S2.

Parental sequence variants can be used as genetic markers of viral transmission at the single-cell level. Several of these variants were found in more than one cell, such that in total 73 of the 90 cells contained at least one (Figure 2A). Of these, 41 cells (56%) contained multiple parental variants (Figure 2B). This was incompatible with the MOI used, according to which only 15% of the cells should be infected by more than one particle (each particle should contain one viral genome; Green et al., 2006). Some viral genomes present in the inoculum may harbor multiple mutations, but since these would be genetically linked, they should be found in the same plaques derived from each cell. However, only three pairs of variants were associated in that manner (C4804U and G10240A in cell 12, G4249A and A8671G in cell 37, G3871A and A4983C in cell 79), therefore indicating that the elevated number of parental variants entering some cells was not explained by genetic linkage. Since VSV shows an extremely low rate of recombination (Han and Worobey, 2011) and the MOI used was relatively low, the possibility that parental sequences were systematically reshuffled after a single infection cycle can also be rejected. We also found that, on average, parental variants were found in only 51% of the plaques derived from individual infected cells (Figure 2C). However, if most cells were infected by a single particle, these variants should be typically found in all viral progeny derived from individual cells. Therefore, this observation further supports the conclusion that a large fraction of the cells were infected by several particles. Similar to many other RNA viruses, classical electron microscopy studies provided evidence that VSV exhibits a high viral particle-to-PFU ratio and that individual PFUs are often constituted by aggregates of infectious viral particles (Arstila, 1976; Galasso, 1967). In combination with these structural properties, our results reveal that multiple infectious particles contained inside individual infection units (PFUs) can deliver pre-existing viral genetic diversity to individual cells, even at low MOI.

### Allele Co-transmission within Single Infectious Units Can Determine the Viral Phenotype

**Figure 3:**
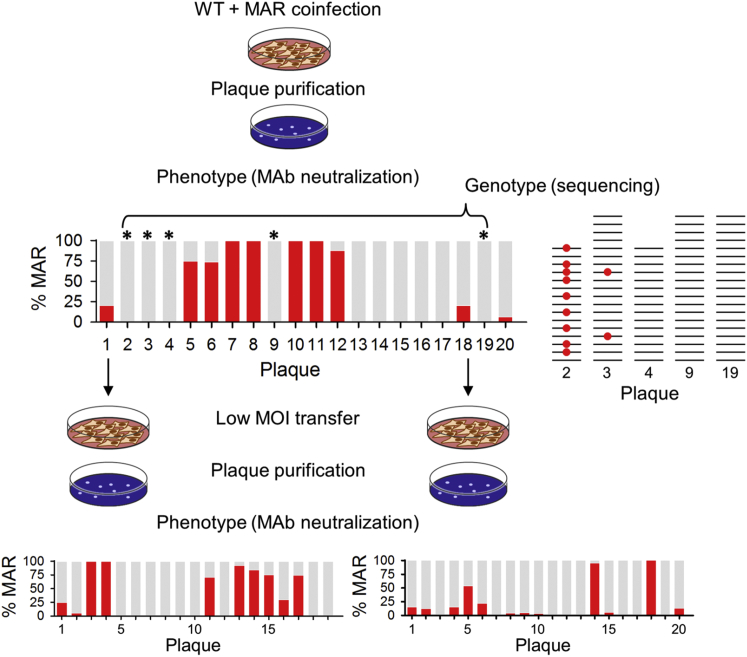
Co-transmission of Sequence Variants BHK cells were co-infected (total MOI = 20 PFU/cell) with the wild-type (WT) and a MAR variant. After completion of the infection cycle, progeny viruses were used for plaque purification, and 20 plaques were picked. Each of these plaques was then assayed for its ability to escape antibody neutralization by plaque assays in the presence or absence of a monoclonal antibody. Red histogram bars show the relative proportion of MAR viruses within each plaque as determined by the neutralization assay. Plaques 1, 5, 6, 12, 18, and 20 showed a mix of MAR and WT phenotypes. Five plaques with fully WT phenotype (2, 3, 4, 9, and 10; marked with an asterisk) were further analyzed by RT, PCR, molecular cloning and Sanger sequencing of 15–19 clones each. Plaques 2 and 3 showed a mix of the WT sequence and MAR-conferring mutations, despite their fully WT phenotype. Plaques 1 and 18 were subsequently used for infecting fresh BHK cells at 0.02 PFU/cell and, after the entire culture was infected, 19–20 plaques were isolated and used to repeat the neutralization assay. Mixing of MAR and WT viruses was still evident after this additional transfer.

To more directly test whether genetic variation could be co-transmitted within individual PFUs, we infected BHK cells with a 1:1 input ratio of the wild-type VSV and a monoclonal antibody-resistant (MAR) mutant at a total MOI of 20 PFU/cell to allow for extensive co-infection of cells with these two variants in the first infection cycle. Viral progeny were harvested after 24 hr, and 20 individual plaques were then isolated (Figure 3). Cross-contamination between plaques was discarded because few or no viruses were detected in 20 spots sampled randomly in between plaques (< 10^2^ PFU/ml versus > 10^7^ PFU/ml in plaques). If PFUs were constituted by single viral particles, they should represent clones of either fully wild-type or fully MAR phenotype, whereas if each PFU contained several co-transmitted particles, it should be possible to find both wild-type and MAR viruses within the same plaque. Neutralization assays revealed a fully wild-type, antibody-sensitive phenotype in 10 of the 20 plaque-purified viruses examined, a fully MAR phenotype in 4 cases, and a mix of both phenotypes in 6 cases, the later indicating co-transmission of the two variants. We further examined 5 wild-type plaques by RT-PCR, molecular cloning, and Sanger sequencing of 15–19 clones from each. Strikingly, in two of these plaques with fully antibody-sensitive phenotypes, we found 9/15 and 2/19 sequences containing previously described MAR mutations (Combe and Sanjuán, 2014). Therefore, in total, 8/20 plaques showed evidence for co-transmission of the two alleles within the same PFU. However, as determined by the neutralization assay, in some cases MAR mutations were unable to confer antibody escape to the virus and thus behaved as recessive alleles. This result shows that the effect of a given mutation can be determined collectively by members of multi-particle infectious units. To test the stability of allele co-transmission, we performed an additional transfer at low MOI (0.02 PFU/cell) of two of the plaques showing mixed phenotypes, and we repeated the plaque purification step. In each of these two cases, the presence of the MAR and wild-type phenotypes was still evident after this additional transfer, indicating that their co-transmission lasted for several generations.

### VSV Genetic Diversity Is Rapidly Replenished after Single-Cell Bottlenecks

**Figure 4:**
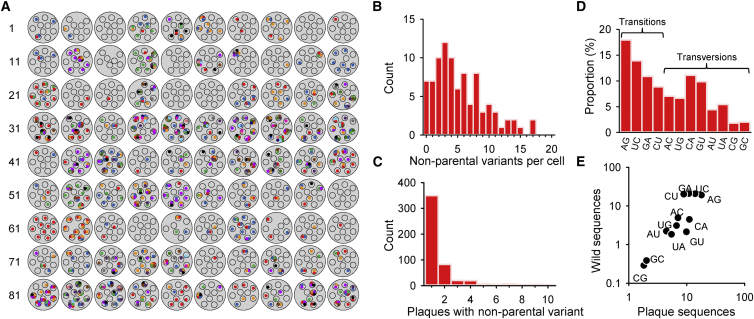
Genetic Diversity Found after Single-Cell Bottlenecks (A) Non-parental SNPs found after single-cell bottlenecks. Large gray circles depict individual cells indicated with numbers and smaller circles correspond to the 7–10 plaques derived from each cell. The 496 SNPs are shown with colored dots inside each plaque. Colors are used for distinguishing different SNPs found among plaques derived from the same cell. Use of the same color for different cells is not meant to indicate a common sequence variant. (B) Distribution of the number of non-parental SNPs found in the 7–10 plaques derived from each cell. (C) Distribution of the number of plaques derived from the same cell that contained a given non-parental variant. (D) Spectrum of nucleotide substitutions found after single-cell bottlenecks. (E) Correlation between the abundance of each type of substitution in single-cell-derived plaques and natural isolates. For natural isolates, 1,033 SNPs were extracted from the following available genome sequences of the Indiana VSV serotype: EU849003 (Mudd-Summers strain, used as reference), AF473865 (isolated from cattle in Colombia, 1985), AF473866 (isolated from cattle in Guatemala, 1994), AF473864 (isolated from horse in Colorado, 1998), EF197793 (unknown host), J02428 (unknown host), and NC_001560 (unknown host). The dots indicate the percentage of total SNPs of each type (A→G, U→C, and so on), in log scale. The list of non-parental SNPs is provided in Table S3.

The 496 SNPs that were not detected in the inoculum (Table S3) probably represent spontaneous mutations produced shortly after the bottleneck, while some may be low-frequency parental variants not detected by ultra-deep sequencing. On average 5.51 such SNPs were found among the 7–10 plaques derived from each cell, but this number varied from 0 to 17 depending on the cell (Figures 4A and 4B). Base transitions represented 51.6% of these SNPs, the most frequent being A→G (17.9%) and its complementary U→C (13.9%) substitutions, whereas the inverse substitutions G→A (10.9%) and C→U (8.8%) were less abundant (Figure 4D). Among transversions, C→A (11.1%) and its complementary G→U (9.9%) change were the most common, whereas G→C/C→G changes were the rarest (2.0% and 1.8%, respectively; chi-square test: p < 0.001). These frequencies correlate with the spectrum of SNPs found in genome sequences from wild VSV isolates (log-scale Pearson’s correlation: *r* = 0.899, p < 0.0001; Figure 4E), suggesting their relevance to natural viral diversity. The fact that the estimated number of new SNPs produced within two viral generations following the single-cell bottlenecks was over an order of magnitude higher than the number of parental variants infecting these cells (496 versus 36) underscores the remarkable capacity of RNA viruses to rapidly regain high diversity. Of the 496 SNPs, 338 (68.1%) changed protein sequence (nonsynonymous), 149 (30.1%) were synonymous, and 9 (1.8%) fell at intergenic regions. These proportions indicate the presence of purifying selection against protein sequence changes since, in the absence of selection, 24.5% of all possible nucleotide substitutions falling at coding regions should be synonymous given the observed ratio of transition/transversions (chi-square test: p < 0.001, dN/dS = 0.737). Therefore, to estimate the rate at which mutations are produced, a correction for selection needs to be implemented. Using the empirically determined distribution of mutational fitness effects as described in previous works (Sanjuán et al., 2004; Sanjuán et al., 2010), we estimate that approximately 40% of the spontaneous mutations should be counter-selected within three viral generations (the initial single cell and two subsequent infection cycles for plaque development). Therefore, given that 496 SNPs were observed in 881 full-length (11,161 bases) sequences, the estimated mutation rate is 496/881/11,161/3/0.6 = 2.8 × 10^−5^ mutations per nucleotide per cell infection, a value very similar to previous estimates obtained by different methods (Sanjuán et al., 2010).

### Viral Yield Correlates with the Early Production of Genetic Diversity

**Figure 5:**
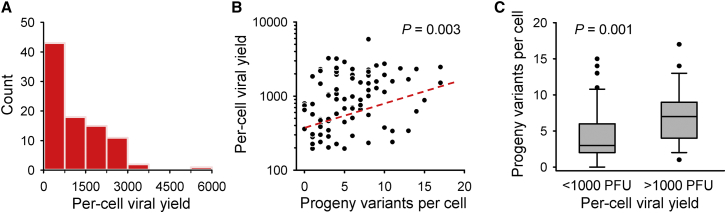
Relationship between the Viral Yield per Cell and the Genetic Diversity Produced Immediately after Single-Cell Bottlenecks (A) Distribution of the viral yield among the 90 cells. (B) Correlation between the per-cell viral yield and the number of non-parental sequence variants found among the 7–10 plaques derived from each cell. The red dashed line indicates the linear regression line. The indicated p value rejects the null hypothesis of a null slope. (C) Box plot of the number of new variants found among the 7–10 plaques derived from each cell as a function of the per-cell viral yield with a 1,000 PFU/cell cutoff. Lines within each box mark the median, the boundaries of the box indicate the 25^th^ and 75^th^ percentiles, whiskers indicate the 10^th^ and 90^th^ percentiles, and dots show individual outlying points.

The number of mutations found in the 7–10 plaque sequences derived from each single-cell bottleneck was over-dispersed compared to a Poisson distribution (variance to mean ratio: 3.1, Kolmogorov-Smirnov test: p = 0.003; Figure 4B), indicating that some cells produced significantly more diverse viral progeny than others. The production of spontaneous mutations should depend not only on their probability of appearance, but also on the number of rounds of copying within each cell. Our data provide evidence that VSV undergoes multiple rounds of copying per cell because, although most of the 496 mutations were unique, 147 were found in two or more plaques derived from the same cell, showing that the mutation was copied during viral replication within the very first infected cell (Figure 4C). Undergoing multiple rounds of copying should allow viruses to produce progeny more efficiently because the genetic material is amplified geometrically. However, this should also produce a greater number of mutations. To test this expected association, we used the viral yields of each of the 90 initially infected cells. The average per-cell yield was 594 PFUs, but showed extensive among-cell variation (Figure 5A). We found a significant correlation between the number of mutations in the viral progeny and the log-yield of the initially infected cell (Pearson *r* = 0.302, N = 90 cells; p = 0.003; Figure 5B). Similarly, viral sequences derived from cells producing more than 1,000 PFUs tended to show slightly more SNPs (6.97 ± 0.67; N = 38 cells) than those from cells with lower yields (4.29 ± 0.52, N = 52 cells; Mann-Whitney test: p = 0.001; Figure 5C).

### Single-Cell Viral Progeny Shows Variable Rates of Spontaneous Mutation

Another possible mechanism that may determine the rate at which a virus replenishes diversity after a bottleneck is the presence of mutators, which have been previously shown to significantly contribute population adaptability in bacteria (González et al., 2008; Jolivet-Gougeon et al., 2011; LeClerc et al., 1996; Sniegowski et al., 1997) but not in RNA viruses. Cell 36 showed the greatest production of genetic diversity, with 17 SNPs not found in the inoculum (i.e., non-parental). This cell was infected by a virus carrying the parental sequence variant A6150C, which produces a N473T amino acid replacement in the viral replicase L protein. However, this variant was only transmitted to one of the ten sequenced plaques derived from cell 36 (plaque 36.2). Additionally, plaque 36.2 showed a second, non-parental replacement in the L protein (M957V), which probably appeared during infection of cell 36 or the early growth of plaque 36.2, and which mapped to the conserved region (CR) IV of the protein, a key structural component of the replicase ring domain (Rahmeh et al., 2010). To test whether viruses derived from this plaque showed changes in the rate of spontaneous mutation, we performed Luria-Delbrück fluctuation tests using a monoclonal antibody to score MAR mutants, as described previously (Combe and Sanjuán, 2014). For comparison, we selected two other plaques from cell 36 and two plaques from cell 81, which showed a lower level of viral diversity, with only one non-parental sequence variant. We found differences in the rate of spontaneous mutation among plaques (one-way ANOVA: p = 0.002, Table 1), with plaque 36.2 showing the highest rate and a significant 3.6-fold increase compared to the other four plaques (Tukey’s post hoc test: p < 0.05). These results suggest that heterogeneity in the rate of spontaneous mutation, as determined by changes in the polymerase sequence, contribute to explaining differences in the ability of VSV to produce genetic diversity shortly after transmission bottlenecks.

## Discussion

Ultra-deep sequencing of viruses very few generations after single-cell bottlenecks allowed us to quantify the short-term production of genetic diversity. We found on average 5.51 new SNPs in the full-length sequence of 7–10 plaques derived from individual cells. Since the mean viral yield per cell was 594 PFUs, we estimate that > 300 total new sequence variants were produced in the first three viral generations. Furthermore, pre-existing genetic diversity was also transmitted to individual cells within single PFUs, implying that cells can often be infected with more than one sequence variant of the virus even when the MOI is low. This has important consequences for virus evolution because it promotes the maintenance of genetic diversity even under extreme transmission bottlenecks and creates a previously unrecognized type of linkage between alleles. Following co-infection of the same cells with two or more mutant viruses, viral products can be shared, enabling the genetic complementation of deleterious alleles (Novella et al., 2004). Transmission of viruses as aggregates of infectious particles may also help explaining why genetically defective viruses can persist for multiple generations in nature, as shown for dengue virus (Aaskov et al., 2006). Related to this, it has been shown that poliovirus fitness can be determined by cooperative interactions established among genotypes, thus making viral fitness an emergent property of the population rather than an individual property (Vignuzzi et al., 2006), and our results provide a structural and genetic basis for these observations. Classical electron microscopy studies showed particle aggregation in a variety of viruses in addition to VSV, including influenza virus (Hirst and Pons, 1973) and vaccinia virus (Galasso and Sharp, 1962), and it has been recently demonstrated that poliovirus and other enteroviruses are transmitted from cell to cell as pools of particles confined inside phosphatidylserine vesicles (Chen et al., 2015). However, only a subset of the viral particles in each aggregate or vesicle may be infectious, and a fraction of these may be genetically identical. Our results show, though, that single PFUs harbor multiple, infectious, and genetically non-identical particles and provide quantitative information about their diversity.

We have also found that the amount of genetic diversity produced shortly after single-cell bottlenecks is highly variable depending on the particular cell infected, and we have identified the per-cell viral yield and mutation rate variation as two determinants of short-term diversification. VSV relies on rapid replication and non-specific inhibition of host gene expression to evade innate immunity (Faul et al., 2009). Although immune pressure is probably absent during the very early stages of the infection of each new individual host, the setup of interferon responses mediated by cell signaling can strongly inhibit the replication of VSV and many other viruses shortly after (Hoffmann et al., 2015). At this stage, viral genetic diversity may be critical for immune evasion and virus survival. The finding that cells producing more viral progeny also showed more diversity can be explained by the geometric amplification of viral genomic RNA within cells (Safari and Roossinck, 2014). Although it has been suggested for several RNA viruses that replication proceeds via a “stamping-machine” model in which progeny genomes are all made from the same initial template (García-Villada and Drake, 2012; Chao et al., 2002; French and Stenger, 2003; Martínez et al., 2011; Safari and Roossinck, 2014), recent work with poliovirus (Schulte et al., 2015) suggests multiple rounds of copying per cell with significant stochastic variation among cells, in line with our results.

Finally, we also found that changes affecting replication fidelity can foster the appearance of new mutations during the early stages of viral growth. However, as a result of their greater genetic load, mutator viruses may be short-lived and stay at low frequencies in the viral population. Supporting this possibility, measurement of the global mutation rate of the same viral stock yielded a value very similar to those of plaques 36.1, 36.9, 81.9, and 81.10, and lower than for plaque 36.2 (Combe and Sanjuán, 2014). It is well-known that the extremely error-prone replication of RNA viruses endows them with a remarkable ability to adapt to changing environments (Andino and Domingo, 2015; Duffy et al., 2008; Lauring et al., 2013; Pepin et al., 2010). However, such error-prone replication also situates RNA viruses in the verge of an extinction threshold beyond which the genetic stability of the viral population can be no longer maintained (Manrubia et al., 2010; Sanjuán, 2012). How RNA viruses resolve this paradoxical relationship between mutation and survival is still poorly understood. By clustering genetic diversity in a subset of cells while preserving a more conserved sequence in other cells, viruses may adopt a population structure that is more robust to the deleterious effects of mutations, yet still highly evolvable.

## Experimental Procedures

### Virus and Cells

The VSV stock used here was obtained from a cDNA clone as detailed previously (Sanjuán et al., 2004) and was subsequently passaged in BHK cells (ATCC) as indicated. The sequence of this virus was identical to a previously described sequence (GenBank: AM690336) except for six point substitutions (A3351G, C6190A, A6523G, A7421C, U9532C, and U11136A). Cells were cultured at 37°C under 5% CO_2_ in DMEM (Dulbecco’s modified Eagle’s medium) supplemented with 10% fetal bovine serum (FBS), 0.02 mM L-glutamine, non-essential amino acids, 100 μg/ml streptomycin, 60 μg/ml penicillin, and 2 μg/ml fungizone.

### Ultra-Deep Sequencing of the Inoculum

To detect SNPs present in the inoculum (parental variants), viral RNA was column-purified with NucleoSpin Viral RNA Isolation kit (Macherey-Nagel) and used for high-fidelity reverse transcription with Accuscript according to manufacturer’s instructions (Agilent Technologies). High-fidelity PCR of the full viral genome was performed using Phusion DNA polymerase (New England Biolabs) in four overlapping fragments of approximately 3 kb length each. PCR products were purified with the NucleoFast 96 PCR Plate kit (Macherey-Nagel) and quantified with the Qubit dsDNA HS Assay kit (Invitrogen). The four fragments of each PCR were mixed in equimolar proportion and subject to high-coverage paired-end sequencing in a MiSeq Illumina machine (Figure S1). This process was done in triplicate to minimize PCR amplification biases. To ascertain the sequencing error rate, a purified *E. coli* plasmid DNA was also sequenced in parallel. FASTQ files were cleaned by primer removal using CutAdapt 1.6 and de-replicated, and low-quality sequences were removed using PrinSeq-lite v0.20. Filtered reads were mapped onto the sequence of the VSV stock used for inoculation using the local aligner Bwa. Mapped files were then converted into binary format (BAM), sorted, and indexed in SAMtools. Base coverage across the viral genome was computed with BEDTools. By converting post-alignment BAM files into mpileup format in SAMtools, SNPs were called using VarScan v2.3.7. Analysis of plasmid indicated that Illumina sequencing errors occurred at a frequency < 0.1% (Figure S2).

### Single-Cell Isolation

BHK cells were grown to a confluent adherent monolayer, washed with PBS, detached with trypsin, resuspended in DMEM supplemented with 2% FBS, inoculated with VSV at the indicated MOI, and incubated for 45 min at 37°C to allow for viral adsorption. The inoculated cells were then diluted 1/20 in the same medium and placed in a 60-mm dish to isolate individual cells with a G-1 glass capillary (Narishige) made to fit the diameter of BHK cells and set onto a Narishige MN-151 micromanipulator under an inverted microscope. Individual cells were focused at 100 × magnification, aspirated in the glass capillary, and immediately re-injected in a drop of 10 μl DMEM using an O_2_-supplied Narishige IM-300 micro-injector. Drops containing single cells were pipetted and released into 100 μl of infection media in an assigned well of a 96-well plate, and cells were incubated 24 hr to allow for viral replication and progeny release. Aliquots of the supernatant were then stored at −70°C. The single-cell isolation procedure was repeated to ascertain the possibility of carry-over contamination from viruses present in the inoculum. The number of PFUs per well was clearly bimodal, with either one or zero PFU/well or > 100 PFU/well, thus indicating that < 1% of the plaques could originate from the viral stock used for inoculation (Figure S4).

### Plaque Assays

These assays were used for titration and to isolate single plaques for sequencing or for phenotypic assays. Viruses were diluted and used to inoculate BHK monolayers, which were subsequently incubated for 24 hr in DMEM supplemented with 2% FBS and semi-solidified with 0.4% agarose. Monolayers were used to aspirate individual plaques or fixed with 10% formaldehyde and stained with 2% crystal violet to count PFUs. In MAR phenotype assays, plaque assays were done in the absence and in the presence of a monoclonal antibody against the surface glycoprotein G at a concentration that neutralizes completely the wild-type virus. The antibody, in the form of a hybridoma supernatant, was added to the medium (25% v:v) to avoid phenotypic masking (Holland et al., 1989).

### Plaque RNA Extraction, RT-PCR, and SOLiD Sequencing

For each cell, ten individual, well-isolated plaques were picked from distinct 60-mm dishes to avoid cross-contamination, stored in multiple aliquots at –70°C, titrated by triplicate plaque assays, and equalized to 10^6^ PFU/ml. Given the large number of plaques (881), pools of 7–10 plaques were made for reasons of tractability before RNA extraction, such that each plaque should be represented at roughly 10% frequency in each pool. Each plaque was assigned to a unique pair of pools, the first containing all plaques from the same cell, and the second containing ten plaques from different cells, to allow for plaque calling after sequencing. Viral RNA from each pool was purified and used for high-fidelity reverse transcription and PCR of the full viral genome, PCR products were purified and quantified as indicated above, and the four PCR fragments belonging to the same pool were mixed in equimolar proportions. Then, pairs of pools were made and used for the construction of 90 tagged libraries using the Library Builder Fragment Core Kit (Applied Biosystems), such that the final expected frequency of each plaque within a library was 5%, with each plaque being assigned to a unique pair of libraries. Sequencing was performed in a SOLiD 5500 XL machine (Life Technologies).

### Analysis of SOLiD Data and SNP Mining

Raw sequence data were converted to FASTQ format using the SOLiD System XSQ Tools, 5′ end adapters and PCR primers were removed using CutAdapt 1.6, sequences were trimmed to 75 bases using FASTXTookit v0.014, and PrinSeq v0.20.4 was used for de-replication (removal of identical sequences), selection of reads with a 25–50 base length, trimming of sequences from the 5′ end with base quality < 20, and removal of sequences with ambiguous bases. Filtered reads were mapped onto the sequence of the VSV stock used for inoculation using the local aligner Bowtie 2. Mapped files were then converted into binary format (BAM), sorted, and indexed in SAMtools. Base coverage across the viral genome was computed with BEDTools. The average per-library coverage was 1,794, with a minimum coverage > 100 in 99.7% of the genome (Figure S3). By converting post-alignment BAM files into mpileup format in SAMtools, SNPs were called using VarScan v2.3.7. To rule out sequencing errors, only SNPs with ≥ 10 supporting reads and > 1% frequency in the library were considered. Since each plaque constituted 5% of the pool content, this sets a cut-off frequency of 20% for SNPs within plaques. These SNPs were assigned to libraries according to their tagging, and since each individual plaque was originally assigned to a unique pair of libraries, the presence of a given SNP in a specific pair allowed for its unambiguous assignment to an individual plaque. SNPs appearing at low frequency (< 10%) in multiple libraries corresponding to different cells were removed because they probably represented sequencing artifacts. To further cure parental sequence variants detected by Illumina sequencing of the inoculum, we removed those present in less than 10% of the progeny sequences derived from a given cell, because these pre-existing variants should be found in a large fraction of the single-cell viral progeny, as opposed to mutants produced during the cell infection or during plaque development.

### Fluctuation Tests

These tests were performed as described previously (Combe and Sanjuán, 2014). Briefly, for each test, 32 BHK culture wells of a 96-well plate were inoculated with approximately 300 PFU each (N_i_) and incubated until approximately 3 × 10^4^ PFU/well were expected (N_f_). After a round of freeze-thawing to release intracellular particles, eight cultures were used for standard titration to determine N_f_ and 24 for plating the entire undiluted volume (100 μl) in the presence of an anti-G monoclonal antibody. The mutation rate to the MAR phenotype (m) was calculated using the null-class method, which is based on the premise that the fraction of cultures showing no MAR mutants equals P_0_ = exp[−m(N_f_−N_i_)]. A correction for incomplete plating of the virus was done as described previously (Bradwell et al., 2013). Three independent tests were performed for each assayed virus.

## Author Contributions

Conceptualization, R.S.; methodology, M.C. and R. Garijo; investigation, M.C. and R. Garijo; data curation, R. Geller and J.M.C.; writing, R.S.; supervision, R.S.; funding acquisition, R.S.

## Figures and Tables

**Table 1:** Rates of Spontaneous Mutations Determined by the Luria-Delbrück Fluctuation Test for Plaques Derived from Single Cells

Cell	Plaque	Nucleotide Substitutions	Amino Acid Changes	MAR^a^ Mutation Rate × 10^−5^
36	1	U755A	F231Y (N)	1.08 ± 0.28
36	2^b^	C2833U, G3485U, A7601G	S195F (M), none, M957V (L: CRIV)	4.03 ± 2.02^d^
36	9	A3620C, C4986A, G5450U, G6327U	None, P85H (L), G240W (L: CRI), R532M (L: CRII)	1.27 ± 0.20
81	9^c^	None	None	1.20 ± 0.46
81	10	None	None	0.91 ± 0.15

a This rate refers to the MAR phenotype. To obtain the rate per nucleotide site, this value should be divided by the number of possible substitutions conferring the MAR phenotype.

b This plaque contained the pre-existing substitution A6150C, which produced the amino acid replacement N473T in the L protein.

c This plaque contained the pre-existing substitution A10979C, which produced the amino acid replacement I2083L in the C terminus of the L protein.

d Tukey’s post hoc test p < 0.05.

## Supplemental Information

Document S1. Figures S1–S4

Table S1. List of SNPs Found in the Viral Stock Used for Inoculating Single Cells: Parental Sequence Variants, Related to Figure 2

Table S2. List of Parental Sequence Variants Found in Plaques Derived from Each Cell, Related to Figure 2

Table S3. List of All Other Non-parental SNPs Found in Plaques Derived from Each Cell, Related to Figure 4

Document S2. Article plus Supplemental Information
