# Supplementary material for: Single-Cell Analysis of RNA Virus Infection Identifies Multiple Genetically Diverse Viral Genomes within Single Infectious Units
Source: Cell Host Microbe. 2015 Oct 14;18(4):424–32. doi: 10.1016/j.chom.2015.09.009 (PMC4617633; doi:10.1016/j.chom.2015.09.009)
Supplement: Document S1. Figures S1–S4 [file mmc1.pdf]

Cell Host & Microbe, Volume *18*

Supplemental Information

Single-Cell Analysis of RNA Virus Infection Identifies Multiple Genetically Diverse Viral Genomes within Single Infectious Units

Marine Combe, Raquel Garijo, Ron Geller, José M. Cuevas, and Rafael Sanjuán

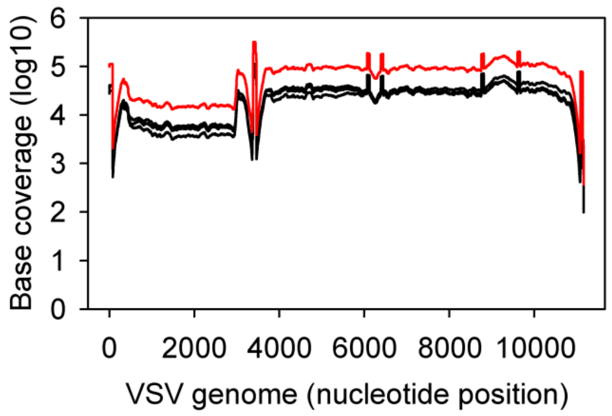

Figure S1 (related to Experimental Procedures). Illumina ultra-deep sequencing coverage throughout the VSV genome. The total final coverage after quality control is shown in red and the coverage for each PCR replicate is shown in black. Since the 3'- and 5'-end primer sequences were removed during the read quality control, the base coverage at the extreme sites of the VSV genome is lowest.

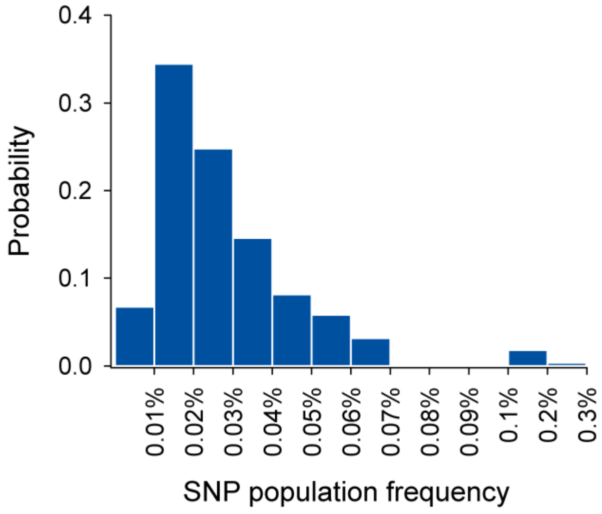

Figure S2 (related to Experimental Procedures). Accuracy of Illumina ultra-deep sequencing for detecting low-frequency SNPs. The distribution of SNP population frequencies is shown for a control DNA consisting of a purified *E. coli* plasmid, which can be regarded as genetically homogeneous in the context of the present study given low mutation rate of the bacterium compared to VSV. Therefore, SNPs detected in the plasmid provide the effective sequencing error rate. Based on this, a 0.1% detection limit was chosen to detect sequence variants in the inoculum.

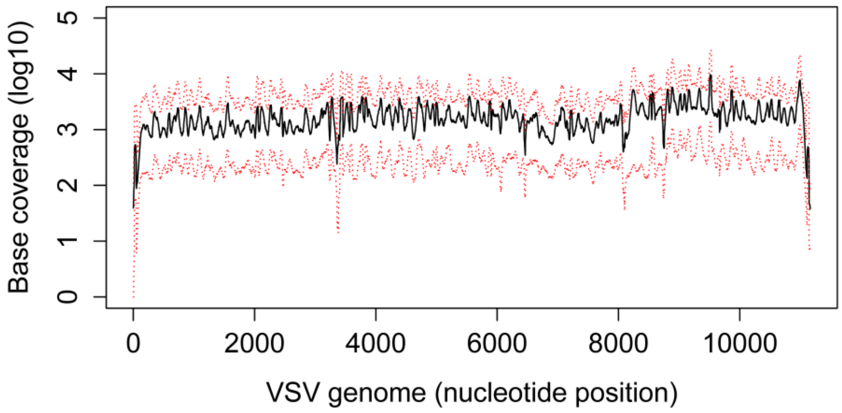

Figure S3 (related to Experimental Procedures). Massive parallel SOLiD sequencing coverage throughout the VSV genome. The final coverage after quality control of the 90 sequencing libraries is shown. The black line shows the mean coverage and the red lines represent the upper and lower base coverage at each position. Since the 3'- and 5'-end primer sequences were removed during the read quality control, the base coverage at the extreme sites of the VSV genome is lowest. The average coverage per library was 1794 reads and 99.7% of the genome was sequenced with >100-fold coverage. Since each plaque represents 5% of a library and was present in two separate libraries, the average coverage per plaque was 179.4 reads and a minimum coverage of 10 reads per plaque was achieved in 99.7% of the genome.

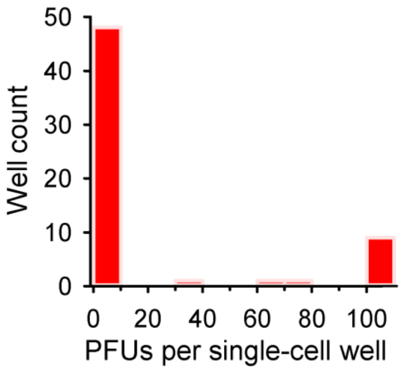

Figure S4 (related to Experimental Procedures). Viral titer per single cell-containing well. The distribution of the viral titer per well is bimodal, with either <10 PFU/well (0 or 1 PFU in all cases) or >100 PFU per well (averaging 594 PFU/well, see text).
